# Prevalence of psychoemotional disorders in patients with pathological kinking of the internal carotid arteries

**DOI:** 10.1192/j.eurpsy.2021.899

**Published:** 2021-08-13

**Authors:** E. Malenkova, O. Zagorulko

**Affiliations:** Pain Clinic, Russian Scientific Centre of Surgery named after B.V.Petrovsky, Moscow, Russian Federation

**Keywords:** Anxiety, Depression

## Abstract

**Introduction:**

Pathological kinking of the internal carotid arteries (PK ICA) is a controversial issue of angioneurology. Patients with PK ICA often present a variety of complaints, such as headache, dizziness, decreased concentration, memory impairment, and general weakness [1].

**Objectives:**

To study the prevalence of anxiety and depression in patients with PK ICA.

**Methods:**

We studied 132 patients who had PK ICA (main group) and 86 patients without brachiocephalic artery pathology (control group). Hospital Anxiety and Depression Scale (HADS) was used to evaluate anxiety and depression, considering depression or anxiety if the score was ≥10. Statistical analysis was performed with SPSS software, p-value<0.05 was considered statistically significant.

**Results:**

The mean age of the patients in the main group was 38.4±5.2 years, in patients of the control group 41.2±4.8 years, respectively. Anxiety disorders were detected significantly more frequently in the main group of patients than in the control group (35.7% and 10.2%, p=0.017 respectively). The frequency of depressive disorders was comparable in both groups – 13.6% and 14.3%, p=0.061, respectively. The level of anxiety was also significantly higher in the group of patients with PK compared to the control group (14.2±4.3 and 9.7±3.1 points, p=0.019). patients with PK ICA with anxiety are more likely suffered from depression (10.2% and 5.8%, p<0.001).

**Conclusions:**

Anxiety disorders were present in one-third of patients with PK ICA, while depressive disorders were not typical for this group. In patients with PK ICA, in addition to collecting complaints, anamnesis, and evaluating the neurological status, it is advisable to conduct neuropsychological testing. References:1.Medvedeva LA, Zagorulko OI.Korsakov Journal 2019

